# A pilot study of an intergenerational program for people in residential aged care with cognitive impairment and children from a co-located early learning centre during COVID-19

**DOI:** 10.1177/14713012241235378

**Published:** 2024-02-19

**Authors:** Nathan M D’Cunha, Helen Holloway, Breanna Cave, Stephanie Mulhall, Annaliese Blair, Katrina Anderson, Daniela Castro De Jong, Susan Kurrle, Stephen Isbel

**Affiliations:** School of Exercise and Rehabilitation Sciences, Faculty of Health, 110446 University of Canberra, Bruce, ACT, Australia; Centre for Ageing Research and Translation, Faculty of Health, 2234 University of Canberra, Bruce, ACT, Australia; School of Exercise and Rehabilitation Sciences, Faculty of Health, 2234 University of Canberra, Bruce, ACT, Australia; Aged Care Evaluation Unit, 37235 Southern NSW Local Health District, Queanbeyan, NSW, Australia; School of Medicine and Psychology, 37235 Australian National University, Acton, ACT, Australia; School of Exercise and Rehabilitation Sciences, Faculty of Health, 2234 University of Canberra, Bruce, ACT, Australia; Centre for Ageing Research and Translation, Faculty of Health, 2234 University of Canberra, Bruce, ACT, Australia; Rehabilitation and Aged Care Services, 638093 Northern Sydney Local Health District, Hornsby, NSW, Australia; Faculty of Medicine and Health, University of Sydney, Sydney, NSW, Australia; School of Exercise and Rehabilitation Sciences, Faculty of Health, 2234 University of Canberra, Bruce, ACT, Australia; Centre for Ageing Research and Translation, Faculty of Health, 2234 University of Canberra, Bruce, ACT, Australia

**Keywords:** intergenerational, cognitive impairment, aged care, nursing home

## Abstract

Intergenerational programs in residential aged care may improve well-being and combat loneliness and social isolation in older people with cognitive impairment. This pilot study investigated the effects of a semi-structured intergenerational group, including children from a co-located early learning centre and people living in residential aged care with cognitive impairment. This 9-week study used a mixed methods pre- and post-program design. Sessions were designed and delivered once per week by Occupational Therapists and took into account residents’ interests and children’s developmental needs and interests, identified in pre-program interviews. Nine older people with cognitive impairment and 13 children participated. The program was well attended despite disruptions and complications caused by COVID-19 and weather conditions. Older people valued the opportunity to engage with the children. Children were observed to gain confidence in communicating and forming friendships with older people with different levels of ability. There did not appear to be any change in loneliness or neuropsychiatric symptoms. The intergenerational program benefited participants and received strong support from family members and staff of the early learning centre and aged care home.

## Introduction

For people living in residential aged care, including the large proportion living with dementia, there is a need for suitable interventions that offer opportunities for engagement in activities that are personally meaningful and promote cognitive stimulation, physical activity and social connection (D'Cunha et al., 2020; O’Rourke et al., 2018; Tierney & Beattie, 2020). An increase in social isolation and restrictions in physical activity may increase neuropsychiatric symptoms, feelings of loneliness, poorer quality of life and cognitive function for older people with dementia living at home but particularly for older people living in residential aged care (Curelaru et al., 2021; Lion et al., 2022). Person-centred group programs are associated with reduced social isolation, loneliness and stress and improved quality of life (D’Cunha et al., 2023a; Manjunath et al., 2021; Quan et al., 2020). In recent years, this loss of community connection among those in residential aged care may have been exacerbated by infection prevention and control measures due to the COVID-19 pandemic (Plagg et al., 2020; Thomas et al., 2022).

Intergenerational programs allow older and younger generations to connect in a group setting while participating in mutually beneficial and meaningful activities, such as reading, arts, crafts, and singing (D'Cunha et al., 2023b). Potential benefits include building connections and relationships between older and younger community members and providing opportunities for social, emotional, and educational growth (D'Cunha et al., 2023b; Golenko et al., 2020; Peters et al., 2021). Engagement in intergenerational programs can contribute to a reduction in age-based stigma and stereotypes as children and young people may develop more positive attitudes toward older people and dementia awareness (D'Cunha et al., 2023b; Di Bona et al., 2019; Galbraith et al., 2015; Gamliel & Gabay, 2014; Gerritzen et al., 2020). Additional benefits for young people include improved communication and language skills, prosocial behaviour (Kessler & Staudinger, 2007), and ability to regulate behaviour (Femia et al., 2008).

It is critical to understand the experience of participants and staff in conducting intergenerational programs. Both older people and parents of young people recognise the importance of community connections and intergenerational relationships with the passing on of knowledge and wisdom between generations (known as generativity) (Kenning et al., 2021; Rosa Hernandez et al., 2020). Barriers to intergenerational programming within residential aged care include staffing, care needs, transportation, and reduced mobility (D'Cunha et al., 2023b). Developing programs that utilise shared or co-located sites, where aged care and childcare are within similar geographical locations, may reduce these barriers.

While some intergenerational studies have included people with dementia and children (Lu et al., 2022), more evidence is needed to inform residential aged care providers and co-located childcare services, on how to develop intergenerational programs for people with dementia and children under the age of 6 before they start attending formal schooling, and what elements make these programs successful (Gerritzen et al., 2020; Kirsnan et al., 2023). This pilot study aimed to investigate the experience of participation in a co-located intergenerational program between a residential aged care home and an early learning centre and includes the perspectives of staff members and program facilitators. The findings may inform future research and enable greater adoption of semi-structured dementia-inclusive intergenerational programming within the residential aged care setting.

## Methods

### Study design

The pilot study was a pre- and post-intervention design. Participants were recruited from the two participating organisations, a residential aged care home and a co-located early learning centre. The study received ethical approval from the Human Research Ethics Committee of the University of Canberra (Project, 202211771).

### Setting

The study was at a single site at a residential aged care home in Canberra, Australia. The residential aged care home has 100 residents and provides personalised care for residents with high care needs, and has registered nursing staff available at all times. The participating children and early learning centre staff walked on a pedestrian pathway to the study site located approximately 100 m away (5-min journey). The program was intended to be outdoors due to COVID-19 safety precautions. Due to poor weather, only one session was completed entirely outdoors, with the on-site community hall used for most sessions.

### Participants

Participants were recruited using purposeful sampling. There were three participant categories in the sample: people with dementia or cognitive impairment who were residential aged care residents and their family member; preschool children attending the early learning centre aged between 4–5 years and their parent/guardian; and staff from the residential aged care home and early learning centre.

The residential aged care activities and lifestyle staff members selected and approached suitable residents they thought might be interested in participating. Researchers contacted the primary family member by phone to schedule an appointment with the potential participant and their family member to explain the study and requested double informed consent. An information session was held for parents at the early learning centre, and those interested in their child participating were invited to contact the researchers and provide informed consent for their child to participate. All workforce staff provided informed consent and were invited to observe each session and support participants. The research team leading the program comprised three occupational therapists who acted as program facilitators, a nurse, and a project manager.

### Study eligibility

Older participants were considered eligible to participate in the study if they were a resident living in residential aged care; willing to provide written consent; had a family member or informal/unpaid care partner to witness their consent and also provide their own written consent and agreement to attend the interviews; could communicate verbally in English; were free from severe hearing or visual impairment as identified by staff report; able to ambulate independently or with assistive devices; have minimal to no history of physical aggression or disruptive behaviour; and have a formal diagnosis of dementia and/or a Clinical Dementia Rating Scale score ≤2 (Morris, 1993, 1997). Children were eligible to participate if enrolled at the early learning centre; available to attend the program weekly; aged between 3–5 years; and consent provided by a parent/guardian on their behalf. One or both parents were also required to consent to participating in pre- and post-program interviews. All early learning centre and residential aged care activity/lifestyle staff involved in helping program participants attend the intervention were eligible to participate in a focus group at the end of the intervention. Residential aged care activity/lifestyle staff also consented to report on their observations of the older people through the study period.

### Intervention

The intergenerational program aimed to promote social skills and interaction through activities incorporating communication, cooperation, and teamwork. For children, developmental outcomes were targeted through activities to promote fine and gross motor skills, remaining focused on task completion or learning new skills. For older people, activities were chosen based on perceived interest and suitability based on physical and cognitive needs. This included familiar activities and those where they could engage in knowledge and skill exchange to increase engagement and ownership in the program. This is in line with the importance of considering meaningfulness and mutual knowledge among the participants as key elements to ensuring successful intergenerational programs (Giraudeau & Bailly, 2019). In order to ensure that these elements were considered, a co-design approach was undertaken with facilitators from the research team and staff from both sites discussing and planning program session structure, and a range of activities before implementation. Tuckman and Jensen (1977) stages of group development were also considered, with earlier sessions aimed at forming the group and building group cohesion, and later sessions which prepared participants for the group to close in a safe and supported way. Older people and family members were interviewed pre-program about their lives to learn about the activities they liked and to identify any concerns using “My Story” (Supplemental Materials 1), a template developed by the American Occupational Therapy Association (American Occupational Therapy Association, 2020). Parents of children also participated in a semi-structured pre-program interview about their expectations and a profile called “All About Me” based on the English version of the Livsboken Guide (Swedish Association of Occupational Therapists, 2011) to understand activities their child enjoyed and identify situations or behaviours to be aware of (Supplemental Materials 2). The information gained through the analysis of these interviews was used to inform the activities within the program

The weekly program typically followed a standard format (Table 1). This planning was arranged by two experienced occupational therapists considering the participants’ occupational preferences, skills, medical history and cultural background. Each session considered the same pattern and was organised following a theme, which would be appealing to all participants (e.g. spring, music, games, etc.). Each session was planned to last for 1 hour as this was deemed the maximum length of time before participants might become fatigued. Before each session, the outline was informed to each facility for their consideration. The program was originally intended to be 8 weeks long. However, due to a COVID-19 outbreak mid-program, one planned group session was cancelled and replaced with a children-only session at the early learning centre. The restrictions related to the outbreak led to cancellation of the session the following week, therefore another children-only activity was planned and all parties agreed to extend the program an additional week.

**Table 1.** Structure of the intergenerational program.

Week	Location	Rational for location	Welcome song	House rules*	Movement activity	Activity one	Activity two	Activity three	Farewell song
1	Outside	Weather and COVID 19 restrictions	“You are my sunshine”	House rules	Parachute (movement)	Getting to know you labels (table top) meet and greet	Treasure hunt (table top) items buried in sand	NA	“Wish me luck as you wave me goodbye”
2	Outside	COVID 19 restrictions and gardening activity	“I like the flowers”	House rules	Spring musical statues	Decorate pots (table top) using stickers and pens	Potting plants (table top)	NA	“Wish me luck as you wave me goodbye”
3	Inside	Weather	“Incy wincy spider”	House rules	House themed musical statues	Building your house and your community use cardboard boxes and decorating	Dress ups	Visiting each others houses	“Wish me luck as you wave me goodbye”
4	Inside/Outside	Sporting activities	“BINGO”	House rules	“Movement bingo"	Pass the parcel	Games rotations outdoor and indoor activities	NA	“Wish me luck as you wave me goodbye”
5	Inside	Weather	“Do Re mi”	House rules	Musical statues	Song list and you tube links	NA	NA	“Wish me luck as you wave me goodbye"
6	Aged care home restrictions in place due to COVID-19. Facilitators spent time with children making messages in a bottle. These were delivered to the older people by staff.								
7	Song/book: We are going on a bear hunt. Aged care home restrictions in place due to COVID-19. Facilitators completed a project with the children.								
8	Inside	Weather	“Jingle bells”	House rules	Dressing up - reindeer antlers and christmas hats for children and residents. Dance themed musical statues	Making our Xmas tree - decorating paper	Building our Xmas tree from the decorated pieces of paper	NA	“Wish me luck as you wave me goodbye”
9	Inside	Weather	“Que será será” and group photo!	House rules	Themed musical statues and favourite song from week one and two	Dress ups	Making and decorating each others certificates	Final event party and certificate presentation	“Wish me luck as you wave me goodbye”

*Notes*. Acknowledgement of Country to pay respect to the land’s traditional custodians was conducted at each session, as required by cultural protocols. House Rules included who to approach if you need assistance; location of the safety station (water, sunscreen and hats, hand sanitiser) and toilets; which areas are out of bounds; expectations - kind, respectful, have a go!; and participation - anyone can sit out any activity.

The three facilitators used the Intergenerational Practice Evaluation Tool (Jarrott, 2019) to reflect upon the program after each session. Staff from the residential aged care home and early learning centre completed an optional written reflection after each session to inform the subsequent weekly program and subsequent adjustments. In weeks six and seven, all non-essential internal and external activities within the residential aged care home were cancelled due to a COVID-19 outbreak. During these two weeks, alternative activities were conducted with children. The alternative activities were oriented to maintain the routines of the program, and focused on engaging remotely with the older participants (e.g. sending them a message in a bottle).

### Measures

Pre- and post-program outcome measures and semi-structured interviews were conducted in-person by team members with older people and a family member (who were interviewed together) (N.D, H.H, and B. C.) and residential aged care activity/lifestyle staff (N.D and H.H). Pre- and post-program interviews were conducted by team members with the parents of the children (N.D, H.H and B.C), and post-program focus groups with early learning centre (S.I and D.C.D.J) and residential aged care staff (N.D and H.H). All team members were experienced conducting interviews as researchers and/or allied health professionals. Pre-program outcomes and interviews were conducted in the two weeks prior to the start of the program. All post-program interviews were conducted within one week of the end of the program. Post-program questionnaires with the residential aged care activity/lifestyle staff were intended to be completed during the same week. However, a COVID-19 outbreak led to a two-week delay.

#### Quantitative

The UCLA 3-item Loneliness Scale is a brief measure assessing three dimensions of loneliness with possible scores ranging from 3–9, with higher scores reflecting increased loneliness (Hughes et al., 2004). This was completed by older participants pre- and post-program. The Neuropsychiatric Inventory Nursing Home Version (NPI-NH) (Wood et al., 2000) is a semi-structured interview containing 12 symptom domains and completed by residential aged care activity/lifestyle staff. Possible scores range from 0–144, with higher scores reflecting greater frequency and severity of neuropsychiatric symptoms. We also scored the occupational disruptiveness questions to assess the impact of neuropsychiatric symptoms on residential aged care staff, with possible scores ranging from 0–60. For the occupational disruptiveness data, residential aged care activity/lifestyle staff consider each of the neuropsychiatric symptoms on a scale of 0–5 with higher scores indicating more disruptive behaviour.

#### Qualitative

Before the program, older participants were asked about their expectations of the program. Post-program, older people were interviewed about their experience, behaviour changes, and emotional reactions to the program or the children. Parents of children were interviewed pre-program about their expectations and any anticipated challenges. Post-program, parents were asked about their observations of their child and their behaviour or reactions. Two focus groups were conducted after the program was complete, one with residential aged care activity/lifestyle staff and one with early learning centre staff. Interviews with the older people were conducted in person, and in-person or online with the parents based on their preferences. The focus groups were conducted in-person. Interview questions can be found in Supplemental Materials 3.

### Data analysis

#### Quantitative

Quantitative data were analysed using descriptive statistics in Microsoft Excel and SPSS Version 27 (IBM, New York, USA). All variables were examined to determine suitability for parametric or non-parametric methods using histograms and Shapiro-Wilk test of normality. Descriptive statistics for normally distributed continuous variables are reported as a mean (±standard deviation) and not normally distributed variables as median values (interquartile range [IQR] Q1 and Q3).

#### Qualitative

Audio-recorded qualitative data collected in interviews and focus groups were transcribed verbatim and de-identified by one team member (H.H) and then checked by two others (N.D and S.I). The data were analysed using a reflexive thematic approach. The analysis used the six-phase process for data engagement, coding and theme development, as Braun and Clarke (2021) described. To reduce risk of bias and enhance trustworthiness and dependability, this was led by two researchers and psychologists (A.B and K.A) who had minimal exposure to the program, undertaking data familiarisation, systematic data coding, generating initial themes from coded data, developing and reviewing themes, refining, defining and naming themes and writing the report (Braun & Clarke, 2021). Two researchers (N.D and H.H) who attended each session checked, queried, and confirmed the results as a form of investiagtor triangulation. Member checking was not performed to reduce participant burden. To maximise best practice for qualitative research, we adhered to the “Consolidated criteria for reporting qualitative research (COREQ)” reporting guidelines (Tong et al., 2007).

## Results

Participant demographics for older people and children are presented in Tables 2 and 3. There were nine older participants with an average age of 91.7 (±3.87), and most were women (77.7%). All older people completed pre-program data collection, and seven completed the post-program interview. Overall program attendance for older people was 92.1%. Six residents attended every session. One participant did not attend the final two sessions following the COVID-19 break in activities and was unavailable for post-program interview. One family carer was not available for a post-program interview. All older people had grandchildren, and three had great-grandchildren. Six had grandchildren who lived in their local area. Overall, participants reported less frequent contact with grandchildren or great-grandchildren after the pandemic started. The education level of older people varied. Seven residents enjoyed activities outside those typically offered by the residential aged care home, including bus trips, drives with family members, and visits to coffee shops or churches.

**Table 2.** Participant information for the older people and family member.

Gender	Age	Length of time living at the aged care home	Highest qualification	CDR (/3)	Family member	Gender of family member
F	94	Less than 1 year	No formal qualification	2	Son	M
F	95	1–2 years	Certificate/Diploma of childcare	0.5	Daughter	F
M	88	1–2 years	Advanced diploma	0.5	Daughter	F
F	87	Less than 1 year	Year 12 or equivalent Diploma not specified	1	Daughter	F
F	97	1–2 years	Advanced diploma	1	Daughter	F
M	93	2–3 years	Diploma	2	Wife	F
F	87	1–2 years	Year 12 or equivalent	2	Daughter and carer who commenced visiting when moved into aged care home	2 × F
F	95	Less than 1 year	No formal qualification	1	Daughter	F
F	92	Less than 1 year	Year 12 or equivalent	0.5	Daughter	F

Key: CDR: Clinical Dementia Rating Scale.

**Table 3.** Participant information for children.

Gender	Age	Age range grandparents (years)
F	5	63–70
NA*	4	58–65
M	5	60–70
M	4	62–67
F	5	62–67
M	4	62–67
F	4	64–72
M	5	58–82
M	4	69–75
M	4	60–90
M	4	55–70
M	4	60–84
F	5	67–77

Key: *NA = Not assigned.

Thirteen children enrolled in the study with an average age of 4.38 (±0.50) years. All parents/guardians completed questionnaires and interviews pre- and post-program. Excluding the two-week program break due to COVID-19, children attended 94.5% of the program, with most attending every week.

### Quantitative outcomes

The mean UCLA 3-item score was 4.14 (±1.35) pre-program and 4.42 (±2.07) post-program. There was a two-week delay after the program finished before the residential aged care activity/lifestyle staff could complete the NPI-NH due to a COVID-19 outbreak. The median NPI-NH score was 15.0 (10.0, 24.0) pre-program and 15.0 (7.00, 42.3) post-program. For NPI-NH occupational disruptiveness, the median was 3.00 (2.00, 7.00) pre-program and 6.00 (4.75, 15.8) post-program. Following removal of one outlier for both scores, median scores post-program were 10.0 (7.00, 30.5) and 5.00 (4.50, 11.0) for the NPI-NH and NPI-NH occupational disruptiveness, respectively.

### Thematic analysis

**Figure 1.**
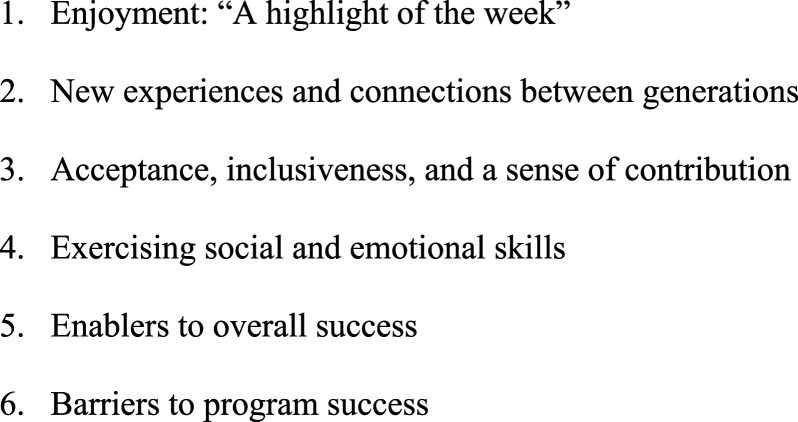
Themes illustrating the experience of participants.

Post-program older people (*n* = 7) and family members (*n* = 8), parents (*n* = 13; with two children having both parents attend interview and one parent with three participating children), as well as early learning centre (*n* = 3) and residential aged care activity/lifestyle staff (*n* = 2) completed interviews. The interviews with participants and their family members lasted between 8.29 and 60.55 minutes, with an average of 28.92 minutes. For parents, they were interviewed for between 4.59 and 16.50 minutes with an average of 10.63. The focus group with the early learning centre staff (*n*-3) lasted 42 minutes, and 34 minutes for the residential aged care activity/lifestyle staff (*n* = 2). Overall, comments from program participants and staff were predominantly positive. This aligned with the expectations of the parents prior to the commencement of the program. There were no major risks, adverse events or insurmountable challenges during program delivery, and all participant groups wished to continue the program at its conclusion. Six themes were identified (Figure 1).

#### Enjoyment: “A highlight of the week”

All participant groups and staff enjoyed the program as it elicited positive emotions. This was reflected by a sense of enthusiasm expressed by staff and parents before the sessions and in their engagement during the session:

…on Mondays, they were sort of excited. It was like, we do exercise in the morning, but they were like “we are, I'm waiting for that program to begin...I might get late.” (Residential aged care activity/lifestyle).

[The child] always looked forward to going on the excursions...When we reminded [the child] of this, that it was happening on Monday morning, [the child] was always keen to go. So yeah, definitely - really enjoyed it! (Parent).

The program allowed residents to enjoy pleasant interactions and facilitated positive ‘in the moment’ experiences through engagement in activities.

She [the resident] was really able to engage…She was able to get in the middle of them, dance, sing. (Residential aged care activity/lifestyle)

[T]he growth…of the residents with their interactions over time...With [resident], when she finally…got up and started dancing, and she started to lead everyone; it was fantastic to see! (Early learning centre)

Program participation appeared to have a lasting impact even when the sessions ended, as residents and children shared what they had done or achieved in the group with others.

And then [older person] yesterday, after she went back, as I said, she had a certificate, and she was showing her certificate to everyone. (Residential aged care activity/lifestyle)

I saw a few residents putting that flowerpot in their rooms. Like, watering every day. (Residential aged care activity/lifestyle)

This impact was identified even where specific details of the program were not remembered by the older person, and residents generally appeared to enjoy being with the children:

I do remember having a good time with the children. Because they’re lovely little children. I just had a good time with them. (Resident)

…we have few residents from [the] dementia [specific unit]. …after doing that activity when I took her back to her unit, I saw her communicating with me with smiles, maybe the activities might have impact her in a positive way. Usually, she doesn't speak or talk with a smile. (Residential aged care activity/lifestyle)

Participation in the program also appeared to impact staff positively and improve their sense of enjoyment and role fulfilment:

…the project also made me feel different when I came back after every session. So, I was full of energy, I was looking forward to it, I was always being happy after the session, I was feeling that I have done something great for someone else. (Early learning centre)

I really get a lot out of it seeing those residents’ happiness, [a] smile on their face, because some of those residents don't smile. (Residential aged care activity/lifestyle)

#### New experiences and connections between generations

Participation in the program appeared to provide residents and children with a unique opportunity to get away from the confines of their usual environment, broaden their social world and break down barriers developed due to participant disability, age or the COVID-19 pandemic.

This got them out of their room and [gave] them a purpose. (Residential aged care activity/lifestyle)

[It’s] going outside that centre, which she does spend eight hours a day, 5 days a week [in]. So, it’s good in that sense as well; just enrichment. (Parent)

I guess some of those residents might not get to engage with young people that often, especially over the last couple of years, so that would be really nice for them. I mean, I see them, you know, smiling and things in the photo. (Parent)

Engaging in the program with children gave older participants a sense of connection to others and the wider community.

It was lucky to have that with the young kids, though. And feel that you are still part of the world. I suppose I am getting to the stage where I won’t be able to do that. (Resident)

Why did I enjoy that? Probably because it was a completely different environment, different people and a change from the usual. (Resident)

Both children and older people appeared to become more relaxed and connected over time, with conversation and spontaneous contact observed as the program progressed, demonstrating that age or disabilities were not a barrier, even among children who initially appeared shy or reserved:

I think that little girl sitting on [older person]'s knee. That was that was really nice. And they held their hands. Some of them even got cuddles. (Residential aged care activity/lifestyle)

They were on the other sides of the table and right in front of them and they were just holding the hands on the top of the table. I thought there was no one encouraging [them] to do that actually. And it was just the conversation flowing away…So, I think that was when I said, the connection is there.” (Early learning centre)

While intergenerational connections were expected to form through program engagement, positive interactions between residents were also reported.

I liked meeting with the children and also leads me to other adults. (Resident)

I just wanted to add that in the program [the resident] met other residents she never met before…She was talking about children, for them both a new experience and plus she met someone who she knows from the past. (Family member)

Among older participants, the group experiences were also thought to promote reminiscence and reflection about previous life experiences:

I think it might be the second day or something with [older person], he was talking to [researcher], and he was reminiscing about the days of old, when he was a teacher, and he was like ‘I used to be a teacher and this reminds so much of when I was a teacher all the children running around’. I thought that was so wonderful. (Early learning centre)

Well, it takes you back to when you’re their age. You see the world sometimes through their eyes when you are mixing with them. (Resident)

Many children did not have regular or close contact with older people. Parents noted connection with others who have differences in ability as a benefit of participation, and staff hoped that this might help children increase their acceptance of older people more generally.

I think just learning about different abilities. I think [the child] understood probably a lot more that [the child] doesn’t normally see. Like my parents, [the child’s] grandparents are fit and well, and just talking to [the child] about ageing and getting old and hearing and mobility and, you know, I think [the child] understood that a lot, you know, [the child] can see that a lot clearer now, I suppose. (Parent)

I think they’ll have it in their memory bank in the back of their head. …I think if they see someone elderly, instead of being actually frightened and hesitating somebody in a wheelchair, they might actually smile at them or say hello [and] not be so frightened, because that’s pretty scary stuff for a little kid. (Residential aged care activity/lifestyle)

#### Acceptance, inclusiveness, and a sense of contribution

The intergenerational program and the social relationships developed were marked by mutual inclusiveness and acceptance. Before participation, parents and facilitators expressed curiosity about the reactions of children to older people and those with differences in ability or physical appearance. During the sessions, children were observed to be accepting of older people, including those with noticeable physical differences or other challenges, such as hearing loss:

They [the children] understood inclusion as well. They were all different residents; [one] of them having oxygen, the residents [in] wheelchairs, and they understood that. We don’t need to tell them, they see it and they understand it, that they are different...they are capable and able to be part of a group, no matter what condition...So, I think that’s a really huge step for them. (Early learning centre)

[The resident] was really hesitant to go because she said nobody will understand her and ‘they'll think that I'm stupid’. But by probably the fourth one, she was just, she did not mention her hearing…she really came out of her shell. I reckon [she] got a lot out of it. She didn't care at the end if they couldn't hear. I was really watching her yesterday, and she was just having a great time. One of the little children came and held her hand, like she thought it was great that they put a wig on her. (Residential aged care activity/lifestyle)

Older participants also expressed a desire to ensure that the children felt included and accepted by following the children’s agenda and joining in on their play.

The children know what they want to do. And they sort of tell you what to do that, or if you want to do that, or look at this, look at this, you know. I like to just join in what they are thinking, and what they might like to talk about, rather than talking down to them. I try not to talk down to them. I have had a few children in my day. (Resident)

Participation in the group allowed residents opportunity to feel they were contributing and passing on knowledge and experience:

Oh, the craft ones, where we built the houses and paper aeroplanes and things like that. Because you really feel you can pass something on to them. (Resident)

I think they all, I think they all enjoyed it. Because we were in a position of giving and not taking, I think, when you are with kids like that. When you’re in there [residential care], you can’t help it, you are taking all the time. (Resident)

#### Exercising social and emotional skills

Staff observed benefits for children, including social skills and assertiveness evidenced by prosocial behaviour within the group setting, such as empathy, sharing, offering help, comfort, and cooperation:

The lady was really struggling getting a sticker off. So, I saw [child], he just gently inserted himself in the situation, helped to get the sticker off and then put it on and then the two of them just like kept going at that point and [they spent a] good five minutes together. So, when it was something like that, where they could visibly see someone who’s struggling, that you could see the children like step in and attempt to help, which is really good. (Early learning centre)

I think what I saw was the group [of] children became so much more empathetic with each other. …For example, someone falls over. Yeah, okay. One child comes over and like, hurts their knee and like another comes over and checks over on them. Or they’ll go and get a teacher or something like that. Now, it’s like, one child hurts, and then it’s a scrum, everyone comes over and checks on them… (Early learning centre)

Children were also observed by early learning centre staff, but not parents, to demonstrate greater capacity for emotional regulation:

I can also see some children’s development in their emotion regulation. Some children can get easily upset in the room for something. Maybe we think it’s small, it matters to them. But I think ever since that child started the project, her emotion regulation has improved so much. (Early learning centre)

This also extended to greater confidence and assertiveness observed among interactions between children and adults:

I think children became a little bit more confident to initiate conversations with older [people], with adults. (Early learning centre)

…some children are becoming more confident. I also noticed this, for example, [child], he was very quiet. I don’t know if being part of this project contributed to when he started opening up. But he started to speak up for himself more. And if something happened to him that he, he didn’t like in the room, he used to just be quiet until an educator notices. But recently, he's been approaching teachers and tell what is going on. (Early learning centre)

#### Enablers to overall success

Participants outlined several enabling factors as key to the program’s success and mitigation of potential or actual challenges. This included close collaboration between staff of partner organisations, choice of activities, careful venue selection and set up, following a set routine, facilitator skill, communication, and use of aids.

With parental permission, early learning centre staff posted photos to an app for parents to view weekly. These photos helped familiarise parents with the program activities and appeared to promote conversation between children and parents about the program.

…having the photos meant that I could say, I saw that you were, you know, painting a box. And then [the child would] say, ‘Oh, yes’, and then tell me a bit more. (Parent)

Similarly, the residential aged care home engaged resident families via photos, which families used to good effect to stimulate memories and discussion with the older person.

I would speak to her in the afternoon and quite often she couldn’t actually remember whether she had been or not. But then later on when I showed her photos, she always sounded positive about it. I don’t remember the specifics she said, but she remembered feeling good about it. And something different and being involved in things. (Family member)

The importance of planning and facilitator skills were identified. Each session ran for approximately an hour, although there was some suggestion that the initial sessions could be shorter while the older people adapted to the program. Good planning, communication, consultation, feedback, and commitment from staff and facilitators were vital to setting up the program and overcoming challenges. This collaborative spirit developed strong relationships between services, which may foster collaboration on other projects.

[W]e needed a partner who was going to be committed and organised as well and it just flowed, what we did, and that is because of you guys. …We were really organised at our end, but together, I think it worked on the day because of the [collaborative] planning. (Facilitator)

I thought they [the facilitators] did a good job. They were very enthusiastic about what they are doing…They did it well and put their hearts into it. (Resident)

The choice of activities was considered vital; they were the glue that brought and held the two generations together. Choosing activities familiar to the older people (e.g., singing familiar songs or working with children for those who were teachers) served two purposes: Firstly, they engaged the older and younger people and made them feel comfortable. Secondly, some of the best activities made older people reminisce about their childhood.

I think the mixture of activities was really good. I think that that sort of creating a bit of routine by starting with a song and things like that, I think that was really helpful. I think having the same people involved running, it was ideal. [The child] remembered. (Residential aged care activity/lifestyle)

And you weren't the only one [resident]. There were a few people who were former choir members who really enjoyed the singing and so that's when I noticed [the resident] really spontaneously joining without needing any one-on- one [prompting]. (Facilitator)

I've seen some children mentioning about the songs that they've been singing. They've been singing them in the room. And they're teaching other children to sing as well. (Early learning centre).

…I heard [child] singing BINGO. Brilliant. And I had never done that with [child]. ...Yeah, sure enough, that's what you guys did that week. (Parent)

The facilitators also used activities to bridge gaps when the group could not meet for two weeks due to COVID-19 restrictions by attending the early learning centre, helping the children send “messages in a bottle” to the residents to maintain a sense of continuity.

Two weeks that you couldn’t participate with the residents' face to face but [the child] really enjoyed the activities that were organised on those days, like sending the letter or the message in the bottle. And he wanted to do that at home to friends, which I thought was really cute. (Parent)

So, when they didn’t, when they weren’t able to come over, when the children made those bottles and they sent them down, that was really good…The letters…some of them are actually hanging up in their [the residents’] rooms. (Residential aged care activity/lifestyle)

#### Barriers to program success

Challenges to sessions taking place were related to unavoidable external events or disruptions, such as COVID-19 which resulted in a temporary suspension of the program or inclement weather which resulted in a location shift. While residents remained on-site for the program, enlisting residential aged care staff in getting residents who required assistance with showering and dressing to be ready for the group sessions was identified as challenging for lifestyle/activities staff:

Getting them over here, that was hard work. [My colleague] and I really worked hard every week because I would put, staff messages out Saturday, Sunday. Maybe one or two might have been ready but we did most of the work”. (Residential aged care activity/lifestyle)

Only one safety incident was mentioned by interviewees; this involved a resident’s oxygen tank becoming accidentally disconnected during the session. This issue was dealt with quickly. Future risk was mitigated by talking to the children about the importance of oxygen for this resident and rearranging the room for future sessions.

## Discussion

This study explored the process and benefits of a structured intergenerational program for people with cognitive impairment living in residential aged care and children from a co-located early learning centre during the COVID-19 pandemic. While the pilot study was not powered to analyse the quantitative results, qualitative findings revealed that the older people, staff, and parents of the children found the program successful and provided meaningful activity for all participants. Several themes were identified in the responses from those involved. Benefits for residents, children and staff were identified, including positive emotions and enjoyment, sense of connection to others, and social and emotional skill development. Staff from the residential aged care and early learning centre observed the older and younger people’s acceptance of each other, the formation of relationships between the older people and children and the benefits received to gradually evolve and compound weekly. During a time in Australia when COVID-19-related restrictions and safety precautions resulted in the suspension or cancellation of intergenerational programs, the program implemented and examined in this study overcame barriers and brought children and older people together again.

Findings from this study are similar to those from previous studies exploring intergenerational programs involving older people and children (Gualano et al., 2018; Holmes, 2009). A novel aspect of the current study was using an early learning centre and residential aged care within walking distance of each other. This decreased the logistical and travel-related barriers to the program and highlighted the value of facilitating intergenerational programming where existing services are located within close proximity. While the use of co-located spaces including both older and younger people has been identified as ideal to facilitate greater intergenerational contact and reduce demands on carers and staff (Jarrott & Lee, 2023) and reduce segregation (Norouzi et al., 2019), findings from this study show intergenerational programs could be conducted within communities where partnerships are formed between organisations located within short geographical distance from one another without any changes to infrastructure or at an organisational level. Despite the lack of travel involved, there is still a need for care coordination to ensure that residents and children are dressed, ready to attend and assisted in walking to the venue.

Implications for practice arising from this study emphasise usefulness of program preparation and design that supports participants’ individual differences, needs, interests, and preferences (Houghton et al., 2022). These elements align with principles of person-centred care in aged care settings (Vassbø et al., 2019) and reinforce the importance of intergenerational activities as a meaningful and enjoyable activity for all involved (Galbraith et al., 2015). In this study, facilitators learned about the participants before designing the weekly sessions which were oriented around discernable themes and included activities connected to participants’ expressed interests and abilities (Galbraith et al., 2015; Vassbø et al., 2019). To our knowledge, the benefit of embedding a structured routine into weekly sessions, which were bookended with group singing, has not been explored in the literature. The program was carefully designed to incorporate intergenerational learning and facilitating occupational therapists reflected upon each session to guide the next. For example, choice and timing of songs helped to start and end each session predictably, provided an atmosphere of comfort and a opportunity for reminiscence. Children learned the songs, and then taught them to other children at the early learning centre who were not part of the program. For older people, memory of familiar music and lyrics can be retained in mild to moderate and even advanced stages of dementia (Cuddy et al., 2012; Garabedian, 2019).

Successful elements of intergenerational programs for people with dementia have been previously identified (Galbraith et al., 2015; Gerritzen et al., 2020) and are further supported by the findings of this study. Activities that facilitate reminiscence are particularly useful as they are less cognitively demanding (Gerritzen et al., 2020) and provide a sense of meaning to older people’s lives (Chung, 2009). As the program activities were designed to suit the level of cognition of the older people, it appears to have reduced the demand for new learning. While some of the challenges encountered in the program’s implementation were similar to those described in the literature (Galbraith et al., 2015; Wendland & Parizet, 2023), we also experienced unique challenges related to COVID-19. Among the similarities to those previously identified was a need for appropriate staff training, which all stakeholders identified, and the level of children’s awareness about older people’s complex health needs and abilities. What could viewed as unique includes the impact of COVID-19 restrictions and outbreak, the weather, and the required time to support older people’s preparation for the session. In a collective case study during the pandemic, Jarrott et al. (2022) acknowledge the need for flexibility in delivering intergenerational programs in the constantly changing aged care environment and strong partnerships among participants and stakeholders. This aligns with the experiences of this study, where facilitators adapted the program for two weeks during the COVID-19 outbreak in the residential aged care home. Despite challenges during the program, no single issue was insurmountable.

### Methodological considerations

The present study was not without limitations. After completion, there were challenges in collecting data from the older people and workforce at the residential aged care due to another COVID-19 outbreak. In addition, two older participants did not complete post-program data collection. Together, these factors may have influenced the quantitative results. We found a small increase in loneliness and a two-week delay in data collection of the NPI-NH meant the ratings included a period of disruption due to a COVID-19 outbreak. In particular, one participant had markedly high scores. The loneliness scores at baseline suggest the participants were not lonely which may explain the absence of a mean improvement using this tool. The group of older participants were diverse in terms of their cognitive and functional impairment, with all but three having questionable or mild ratings on the Clinical Dementia Rating Scale. Therefore, future research should consider recruiting a more well-defined sample to evaluate whether people at different stages of dementia may benefit from the program. This was not possible in our sample as the residential aged care activity/lifestyle staff selected residents they felt would be suitable. We also required double informed consent from both the older person and their family member which limited the pool of residents that were eligible. In addition, as this was a small study, we cannot infer conclusions about whether the cost and time commitment of the program were justified.

Despite the small sample size, there were several strengths. Collecting information about the participants before the program helped the program facilitators customise the program based on the likes and dislikes, and skills of the older people and the children. For older participants with cognitive impairment, including activities, such as singing, that likely activate prior knowledge and skills and reduced the demand and need for new learning. The workforce at the early learning centre and residential aged care home were supported in enabling the participants to engage in the program by the research team, which included highly motivated occupational therapists. Therefore, the results are unlikely to be generalisable to programs with no or limited budget for expenses. Future research may evaluate the use of occupational therapy students to develop a more sustainable model, using learnings from the present study. In addition, there is a need to conduct comprehensive economic evaluations (Vecchio et al., 2021), and evidence of longer-term benefits to participants and the community should be further explored (Lee et al., 2020).

## Conclusion

The intergenerational program was viewed as a successful program by participants, families, and staff from the participating early learning centre and residential aged care. There were reported benefits associated with the intervention between a co-located early learning centre and residential aged care home, despite the need to navigate the ongoing challenges of the COVID-19 pandemic and weather-related complications. Children appeared to build confidence and form relationships with the older participants with cognitive impairment throughout the program. Given that many residential aged care providers know the potential benefits of intergenerational programs but perceived barriers to implementation, more research is needed to increase uptake and develop sustainable models to overcome a challenging workforce and staffing environment in residential aged care. Maximising the use of existing community sites located within close geographical proximity, such as child care and aged care homes, could reduce barriers to intergenerational activities and promote greater social connectivity among older people.

## Supplemental Material

Supplemental Material - A pilot study of an intergenerational program for people in residential aged care with cognitive impairment and children from a co-located early learning centre during COVID-19 Supplemental Material for A pilot study of an intergenerational program for people in residential aged care with cognitive impairment and children from a co-located early learning centre during COVID-19 by Nathan M D’Cunha, Helen Holloway, Breanna Cave, Stephanie Mulhall, Annaliese Blair, Katrina Anderson, Daniela Castro De Jong, Susan Kurrle and Stephen Isbel in Dementia
