# Supplementary material for: A pilot study of an intergenerational program for people in residential aged care with cognitive impairment and children from a co-located early learning centre during COVID-19
Source: Dementia (London). 2024 Feb 19;23(6):927–48. doi: 10.1177/14713012241235378 (PMC11290022; doi:10.1177/14713012241235378)
Supplement: Supplemental Material - A pilot study of an intergenerational program for people in residential aged care with cognitive impairment and children from a co-located early learning centre during COVID-19 [file sj-pdf-1-dem-10.1177_14713012241235378.pdf]

SUPPLEMENTARY MATERIALS

**My Story (**completed **w**ith older person and family**)**
My background<br><br>● Where I grew up (early life: location, who with)<br><br>● Where I lived (adult life; location, who with)<br><br>● My family<br><br>● My working life (occupation, duties, location)<br><br>● Important events in my life<br><br>*Things I really like (ask about TV. Films, books, food, drink, people, clothes, sport cultural activities)*<br><br>*Things I don't like*<br><br>*Things to remember about me*<br><br>*What people say about me*<br><br>*What is important to me*:<br><br>When I meet the children<br><br>What I am looking forward to<br><br>What I am worried about (if anything)<br><br>How would you like the children to greet you (if anything specific)

Supplementary Materials 1. My Story profile questions for older people

Supplementary Materials 2. All About Me profiles questions for children

**All About Me (completed by parent/guardian)**
My name is ______ and I am _________ years old.<br><br>My parent's names are:<br><br>I live with:<br><br>My cultural background is:<br><br>The language we speak is:<br><br>Special events celebrated at home: (e.g., Christmas)<br><br>My favourite things are:<br><br>I love to do the following activities:<br><br>I don't like the following sensations: (e.g., loud noises, bright lights, certain smells/textures)<br><br>If I get upset, it helps if the adults around me…<br><br>Is there anything else important people need to know about me?

Supplementary Materials 3. Semi-structured interview questions

Pre-program interview questions for child parent/guardians

1. What are your expectations for the program?
2. Do you think your child will enjoy meeting and working with the older people? Why/Why not.
3. What do you think your child might get out of the group?
4. Do you think there will be any challenges for the group as a whole?
5. Do you think there will be any problems due to the older people in the group having memory problems?

Post-program interview questions for child parent/guardians

1. Do you think your child enjoyed meeting and working with the older people? Why/Why not.
2. What do you think were the best things about the groups?
3. Was there anything different about your child during the last 9 weeks that you could attribute to the program?
4. Were there any challenges that you are aware of?
5. Based on your knowledge of the groups, if you were to change three things about the program what would they be and why?
6. Would you like your child to continue in the program if it were to continue?

Post-program interview questions for older people

1. Did you like meeting and working with the children? Why/Why not.
2. What were the best things about working with the children?

3. What were some of the challenges working with the children?
4. Did you enjoy working with children? Why/Why not.
5. What are your thoughts on getting together with your fellow residents and the children over the last 9 weeks?
6. If you were to change three things about the program, what would they be and why?

Focus group questions for staff

1. What did you think of the intergenerational group program?
2. What were the best things about the program?
3. What do you think the older people got out of the program?
4. What do you think the children got out of the program?
5. What were some of the challenges about the program?
6. If you were to change three things about the program, what would they be and why?
7. What are some of your general thoughts on intergenerational programs?
